# Cloning and Expression Analysis of the *BocMBF1c* Gene Involved in Heat Tolerance in Chinese Kale

**DOI:** 10.3390/ijms20225637

**Published:** 2019-11-11

**Authors:** Lifang Zou, Bingwei Yu, Xing-Liang Ma, Bihao Cao, Guoju Chen, Changming Chen, Jianjun Lei

**Affiliations:** 1 Key Laboratory of Horticultural Crop Biology and Germplasm Innovation in South China, Ministry of Agriculture, College of Horticulture, South China Agricultural University, Guangzhou 510642, China; 2 Seed and Developmental Biology Program, Global Institute for Food Security, University of Saskatchewan, Saskatoon, SK S7N 0W9, Canada; 3 Henry School of Agricultural Science and Engineering, Shaoguan University, Shaoguan 512005, China

**Keywords:** Chinese kale, thermal tolerance, *BocMBF1c*, expression, localization

## Abstract

Chinese kale (*Brassica oleracea* var*. chinensis* Lei) is an important vegetable crop in South China, valued for its nutritional content and taste. Nonetheless, the thermal tolerance of Chinese kale still needs improvement. Molecular characterization of Chinese kale’s heat stress response could provide a timely solution for developing a thermally tolerant Chinese kale variety. Here, we report the cloning of *multi-protein bridging factor* (*MBF*) *1c* from Chinese kale (*BocMBF1c*), an ortholog to the key heat stress responsive gene *MBF1c*. Phylogenetic analysis showed that *BocMBF1c* is highly similar to the stress-response transcriptional coactivator *MBF1c* from *Arabidopsis thaliana* (*AtMBF1c*), and the *BocMBF1c* coding region conserves MBF1 and helix-turn-helix (HTH) domains. Moreover, the promoter region of *BocMBF1c* contains three heat shock elements (HSEs) and, thus, is highly responsive to heat treatment. This was verified in *Nicotiana benthamiana* leaf tissue using a green fluorescent protein (GFP) reporter. In addition, the expression of *BocMBF1c* can be induced by various abiotic stresses in Chinese kale which indicates the involvement of stress responses. The BocMBF1c-eGFP (enhanced green fluorescent protein) chimeric protein quickly translocated into the nucleus under high temperature treatment in *Nicotiana benthamiana* leaf tissue. Overexpression of *BocMBF1c* in *Arabidopsis thaliana* results in a larger size and enhanced thermal tolerance compared with the wild type. Our results provide valuable insight for the role of *BocMBF1c* during heat stress in Chinese kale.

## 1. Introduction

Chinese kale (*Brassica oleracea* var. *chinensis* Lei Syn:*Brassica alboglabra* Bailey) is a popular vegetable for its health benefits and taste [1]. Chinese kale, belonging to the *Brassica* genus, is native to China and, currently, is predominantly cultivated in South China and Southeast Asia [1]. Recently, the importance of Chinese kale has generated interest in its nutrition and genetics [2]. In particular, Chinese kale contains high levels of glucoraphanin which may possess cancer prevention properties [3].

Unfortunately, Chinese kale is very sensitive to heat stress, which often leads to decreased yield and quality [4]. Consequently, the main growing season and distribution of Chinese kale is limited to the winter season of South China.

High temperature can disturb the membrane, structure of protein, and chromatin architecture of the plant cells which respond with several signaling pathways to address the stress [5]. Many plant hormones are reported in the heat response of plants, and exogenous application of plant hormones can activate expression of heat stress-related genes thus improving thermal tolerance. For example, applying abscisic acid (ABA) can increase the thermal tolerance level by upregulating *ABA responsive transcription factors* (*ABRFs*)*, heat stress transcription factor* (*HSF*) *A2c* (*FaHSFA2c*), and *heat shock proteins* (*HSPs*) in tall fescue [6]; treatment with methyl jasmonate (MeJA) stimulates the expression of *HSP70* [7]; and exogenous salicylic acid (SA) increases the heat stress resistance of wheat by regulating the accumulation of ethylene (ET) and proline, increasing the efficiency of nitrogen usage [8]. Application of ET and jasmonic acid (JA) can activate thermal stress-related genes by upregulating *ethylene response factor* (*ERF*) *1* in *Arabidopsis*.

In plants, enormous progress has recently been made in understanding of the molecular mechanism of thermal tolerance [9]. In addition to HSF- and HSP-responsive pathways [9], the transcriptional coactivator *Multi-protein bridging factor1c* (*MBF1c*) was identified as a critical regulator for thermal tolerance responses in *Arabidopsis* [10]. In *Arabidopsis*, heat stress can lead to increased expression of *AtMBF1c* and triggers the nuclear localization of AtMBF1c proteins. Nuclear AtMBF1c then function as transcription factors to regulate the downstream SA, trehalose, and ET thermal resistance-related pathways [10]. The *AtMBF1c* is a trans-acting regulatory element which recognizes CTAGA as a potential binding sequence and can regulate 36 genes, including stress-responsive gene *dehydration-responsive element* (*DRE*)-*binding protein 2A* (*DREB2A*) [11]. In addition, overexpression of *AtMBF1c* and its ortholog from wheat can improve the performance of host plants under high temperature [11,12]. Therefore, *MBF1c* is an ideal gene for improving plant thermal tolerance and, thus, increase productivity under heat stress. The expression of the *MBF1c* gene in Antarctic Moss (*PaMBF1c*) can be induced by several abiotic stresses, and overexpression of *PaMBF1c* can enhance heat, cold, and salinity stress tolerance in *Arabidopsis* [12]. However, to date there have been no studies conducted on the *MBF1c* of Chinese kale. In this study, we reported the ortholog of *AtMBF1c* in Chinese kale, termed *BocMBF1c*, and characterized its potential role in regulating heat tolerance in Chinese kale.

## 2. Results

### 2.1. Cloning and Analysis of the BocMBF1c Gene

**Figure 1:**
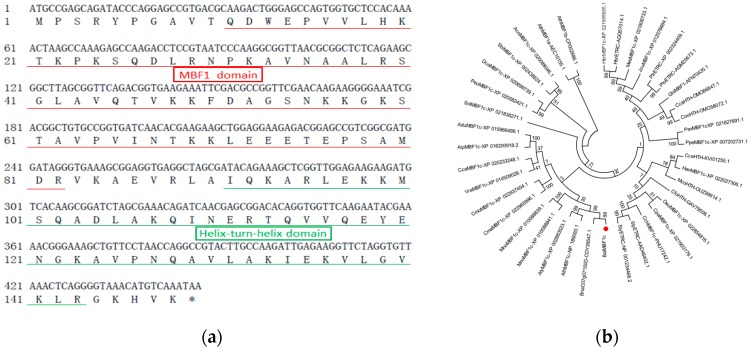
Sequence feature of BocMBF1c protein. (**a**) DNA and amino acid sequence of BocMBF1c. Full-length DNA and deduced amino acid sequence of BocMBF1c. Red underline indicates multi-protein bridging factor1 (MBF1) gene family domain, and green underline signatures helix-turn-helix (HTH) domain. (**b**) Phylogenetic tree constructed by the neighbor-joining method based on the MBF1c amino acid sequences.

As the genome sequence of Chinese kale is still unavailable, we used the *Brassica rapa* MBF1c gene (LOC103828628) as a reference sequence for primer design and then cloned the coding region of *MBF1c* from Chinese kale (variety “Ai-jiao-xiang-gu”) genomic DNA. The resultant amplicon of 707 bp was sequenced and confirmed to be *BocMBF1c* by sequence homology analysis (Figure 1a,b). Open reading fragment (ORF) prediction by ORF finder did not find any intron within the *BocMBF1c* gene, which was then confirmed by agarose gel analysis (Figure S1). Amino acid sequence analysis identified multi-protein bridging factor1 (MBF1) and helix-turn-helix (HTH) domains (Figure 1a). Phylogenetic analysis indicated that BocMBF1c clustered with *Arabidopsis* MBF1c (Figure 1b, Table S1). Next, we obtained the *MBF1c* gene and promoter region of *Brassica oleracea* (Bol013952) by searching the *BocMBF1c* coding sequence (CDS) using BLASTN (http://brassicadb.org/brad/blastPage.php). The 2005 bp DNA fragment containing the promoter of *BocMBF1c* was cloned with primers designed from *BoMBF1c*, from which we used 1409 bp upstream sequence from initiation codon for further analysis. The *BocMBF1c* gene was submitted to GenBank (Accession number: MH685643).

**Figure 2:**
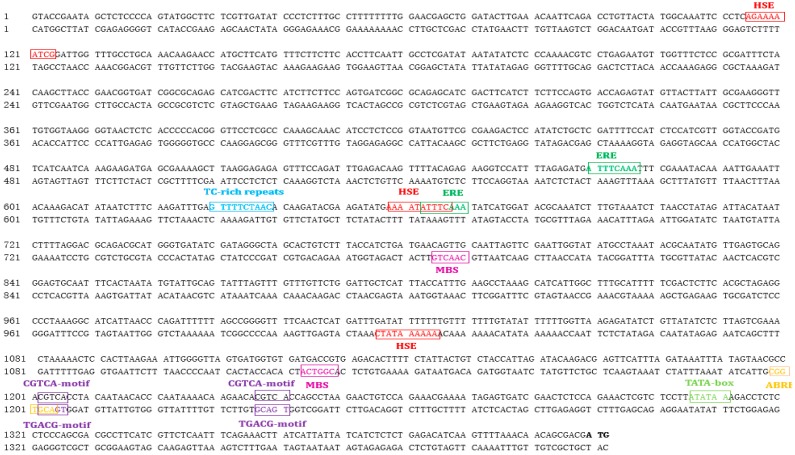
1409 bp upstream of the start code are shown as the *BocMBF1c* promoter. DNA elements in the *BocMBF1c* promoter were designated. The start code is designated as bold and the TATA box as grass green. Three heat shock elements (HSEs) are boxed in red, ethylene-responsive elements (EREs) in green boxes, ABA-responsive elements (ABREs) in yellow boxes, methyl jasmonate (MeJA)-responding elements in purple, MYB binding sites (MBS) in the purple red box, and TC-rich repeats as stress-responding elements in the blue box.

Sequence analysis of the 1409 bp *BocMBF1c* promoter revealed multiple stress and hormonal responsive cis-elements, including heat shock elements (HSEs), ethylene responsive elements (EREs), abscisic acid-responsive element (ABRE), MeJA responsive motifs (CGTCA-motif), drought stress and pathogen-responsive TC-rich repeat, and MYB binding sites (MBS). All of those cis-elements are located upstream of the TATA box (Figure 2).

We cloned the coding region of *BocMBF1c* from genomic DNA, and the deduced BocMBF1c protein contained the *MBF1* gene family domain and HTH domain (Figure 1a and Figure S2). The amino acid sequence of BocMBF1c shared 99% and 94% similarity with its ortholog from *Brassica napus* (CDY39547.1) and *Arabidopsis*, respectively (NP_189093.1). We queried the BocMBF1 protein sequence using the BLASTP program (https://blast.ncbi.nlm.nih.gov/Blast.cgi), and the resulting sequences all belonged to angiosperm. The phylogenetic tree showed *BocMBF1c* was clustered in one group with *MBF1c* genes from dicots, while all *MBF1c* genes from monocots belonged to another group. In addition, *BocMBF1c* belonged to the *MBF1c* gene clade from the Brassicaceae family including *Brassica napus* and *Arabidopsis*. The *AtMBF1a/b* were clustered in an individual cluster, implying they belonged to different members of the *MBF1* gene family (Figure 1b, Table S1).

### 2.2. Transient Expression Analysis Showed BocMBF1c Promoter Activity Can Be Induced by Heat Stress

**Figure 3:**
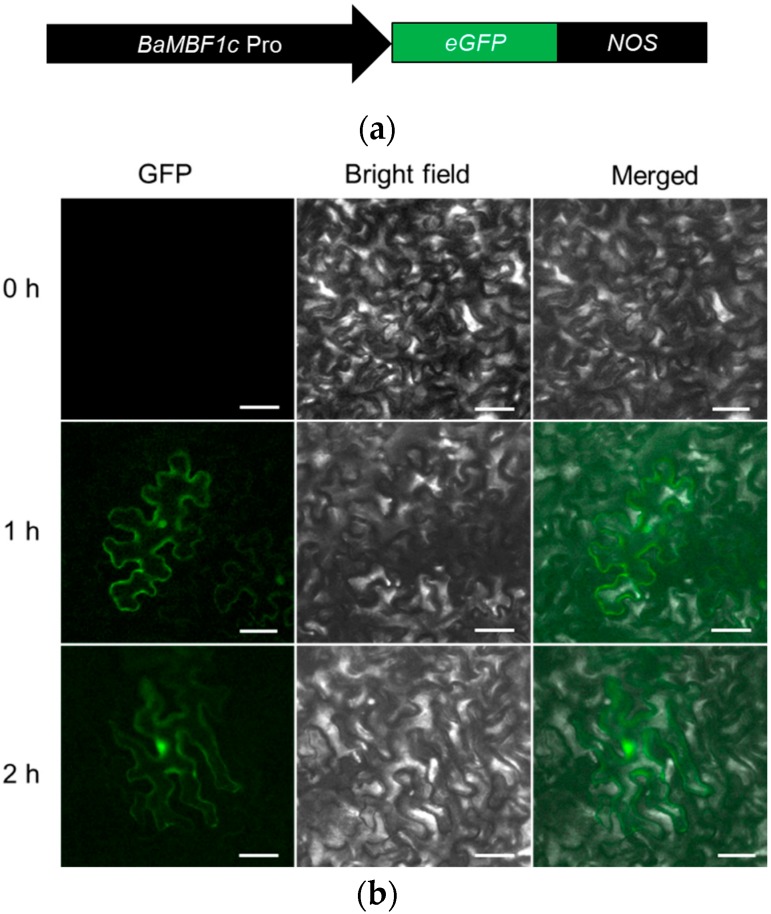
Heat treatment can stimulate *BocMBF1c* promoter activity. (**a**) Legend of pBI101-*pBocMBF1c*-*GFP* construct; *BocMBF1c* promoter was fused with *green fluorescent protein* (*GFP*) coding sequence (CDS) with the *NOS* terminator. (**b**) Treatment at 37 °C induced increased expression in the *Nicotiana* (*N*.) *benthamiana* leaves after agroinfiltration. Scale bar, 50 μm.

The *BocMBF1c* promoter contains several HSEs, indicating its role in heat stress response. Thus, we analyzed the activity of the *BocMBF1c* promoter under heat treatment. We combined the 1409 bp region upstream of the BocMBF1c start code with the CDS of *green fluorescent protein* (*GFP*), and then transiently expressed in *Nicotiana* (*N*.) *benthamiana* leaf tissue by agroinfiltration under ambient and heat-stressed conditions. As predicted, the activity of the *BocMBF1c* promoter was rapidly stimulated under heat stress in Tabaco leaves (Figure 3a,b), which is consistent with previous reports [13].

### 2.3. Expression Pattern of BocMBF1c

**Figure 4:**
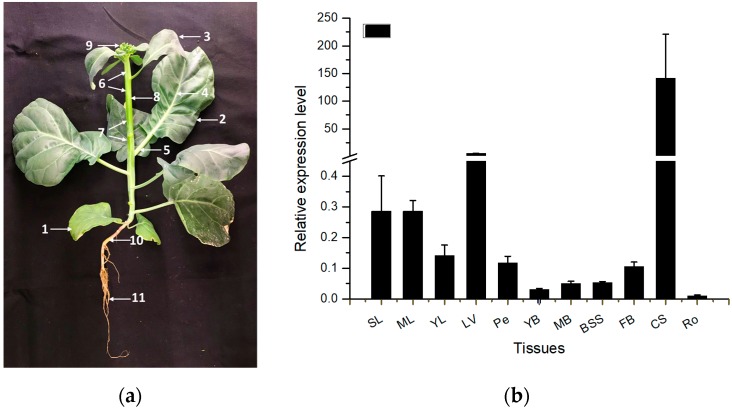
The expression pattern of *BocMBF1c* in different tissues under standard growth conditions. (**a**) Various tissues of Chinese kale. SL, senescent leaf; ML, mature leaf; YL, young leaf; LV, leaf vein; Pe, petiole; YB, young bolting stem flesh; MB, middle bolting stem flesh; BSS, bolting stem skin; FB, flower buds; CS, combining sites between stem and root; Ro, Roots. (**b**) Bolting stage tissues were harvested for expression specificity analysis. Quantitative PCR (qPCR) was used to measure *BocMBF1c* expression in different tissues, with the *BocTubulin8* gene as the control. Data shown are the mean ± SD of three biological replicates.

Under a standard growth environment, expression of *BocMBF1c* was measured in various tissues of Chinese kale (Figure 4a). The results showed *BocMBF1c* had a comparatively higher expression level in combining sites (CS) and leaf veins (LV) and maintained a comparatively low expression level in other tissues (Figure 4b).

**Figure 5:**
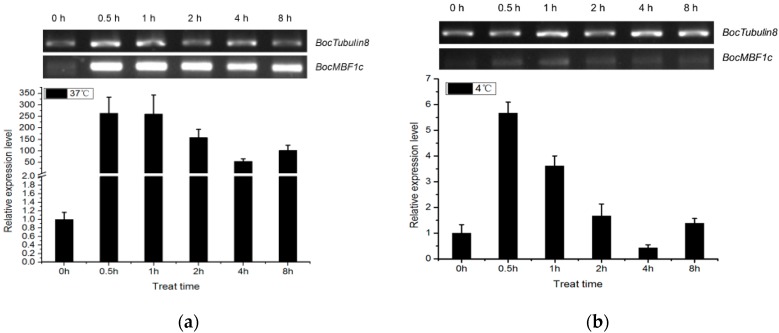

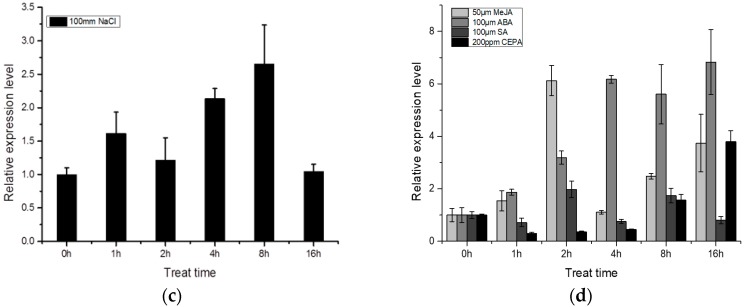
Responses of *BocMBF1c* to various abiotic treatments. (**a**) semiRT-PCR (reverse transcription PCR) and qPCR were used to determine the expression profile under heat stress (37 °C) in the leaf tissue of Chinese kale. (**b**) Expression of *BocMBF1c* under cold treatment (4 °C). semiRT-PCR and qPCR were used to determine the expression profile under cold condition in the leaf tissue of Chinese Kale. (**c**) Responses of *BocMBF1c* to salinity treatment. (**d**) Expression of *BocMBF1c* after applying hormones. All analyses used *Tubulin8* as an internal reference gene, and all data shown were normalized to mock treatments. Data shown are the mean ± SD of three biological replicates.

Under heat stress, *BocMBF1c* expression rapidly increased >250 fold within 0.5 h and maintained this high expression level for at least 8 h in the leaves of Chinese kale (Figure 5a). When the Chinese kale was exposed to chilling conditions, *BocMBF1c* transcript abundance increased five-fold within 0.5 h, then slowly returned to unstressed levels by 4 h post-treatment (Figure 5b). As illustrated in Figure 2, the *BocMBF1c* promoter possessed a series of hormone and other related cis-elements. The qPCR analysis confirmed that *BocMBF1c* was moderately responsive to salinity treatment and several plant hormones including MeJA, ABA, SA, and ET (Figure 5c,d). Interestingly, the temporal response patterns of *BocMBF1c* expression were distinct among different treatments. Under heat and cold stress, the increase in the *BocMBF1c* gene reached full extent within 0.5 h. For salinity treatment, the expression level of *BocMBF1c* reached the maximum after 8 h. When MeJA, ABA, SA, and ethephon (CEPA) were applied, the highest *BocMBF1c* transcription activities were detected after 2 h, 8 h, 2 h, and 16 h, respectively. Overall, our results indicated that other abiotic factors could also influence the transcript level of *BocMBF1c* besides responding to high-temperature conditions. These results indicate that *BocMBF1c* in Chinese kale involves resistance to multiple stresses including heat stress.

### 2.4. BocMBF1c Protein Localizes to the Nucleus under Heat Stress

**Figure 6:**
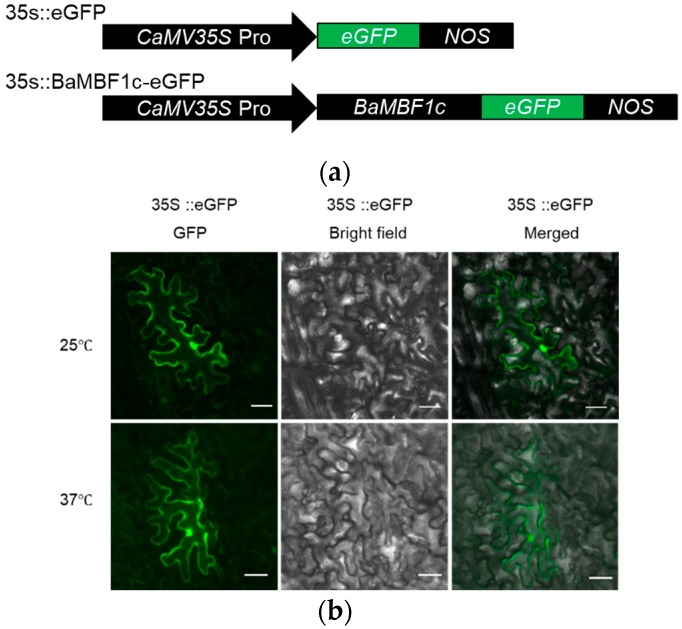

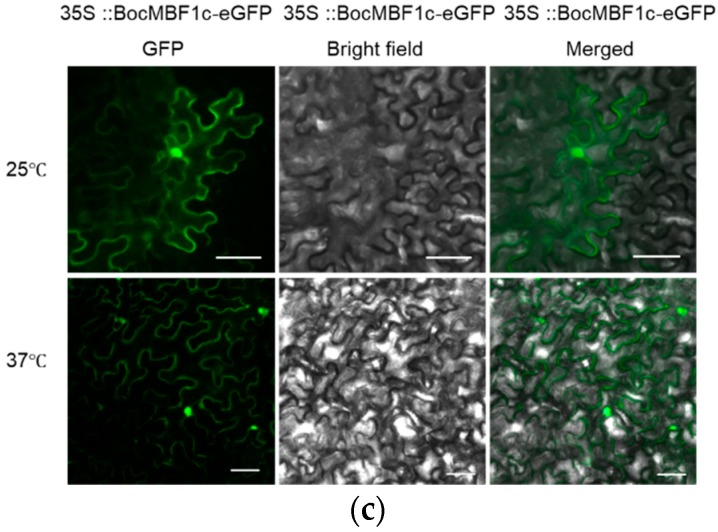
Heat stress led to the nucleus localization of the BocMBF1c protein. (**a**) Schematics of the constructs for 35s::*eGFP* and 35s::*BocMBF1c*-*eGFP* (pBE-*BocMBF1c*-*eGFP*). (**b**) The GFP protein alone has no obvious localization preference either under normal temperature or high temperature in *N. benthamiana* leaf tissue. (**c**) BocMBF1c translocated to the nucleus when exposed to 37 °C for 1 h. Scale bar, 50 μm.

Under normal temperature, the GFP-tagged BocMBF1c protein shows no obvious distribution preference when transiently expressed in *N*. *benthamiana* leaf tissue (Figure 6b). However, the chimeric BocMBF1c-eGFP protein quickly concentrated into the nucleus when exposed to 37 °C for 1 h (Figure 6c). This translocation is critical for the functionality of this heat responsive transcription coactivator.

### 2.5. Phenotype and Thermotolerance Analysis of BocMBF1c Overexpression Lines

**Figure 7:**
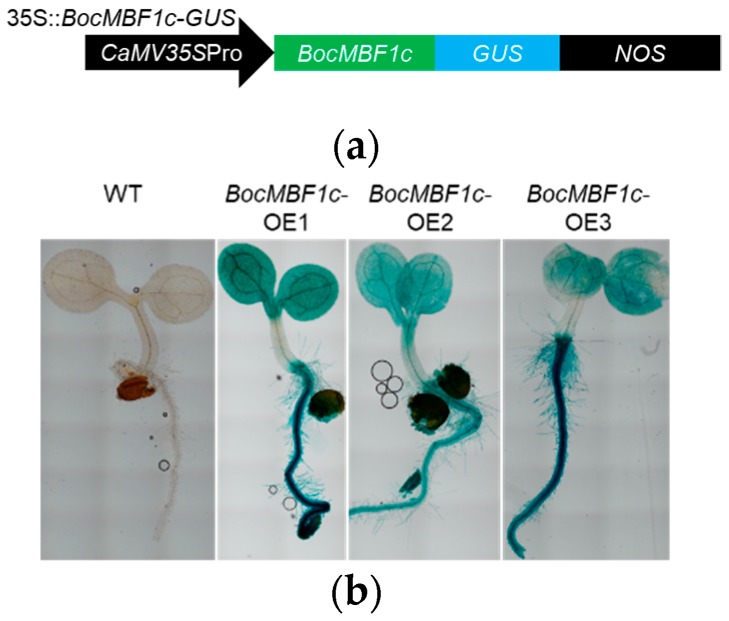

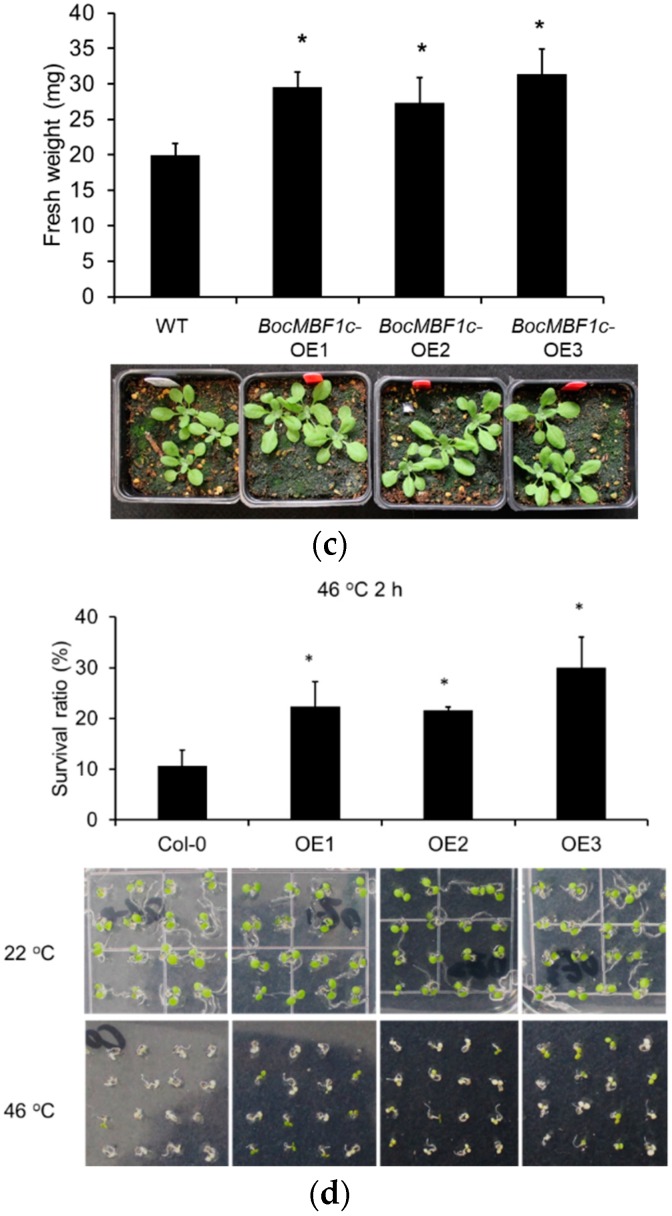
Phenotypic characterization of *BocMBF1c-OE* plants. (**a**) Schematic illustration of the 35s::*BocMBF1c-GUS* construct used for ectopic expression. (**b**) Expression of *BocMBF1c* was validated by β-glucuronidase (GUS) staining (*n* ≥ 15). (**c**) Compared with the wild type (WT) seedlings, the fresh weight of the *BocMBF1c*-OE plants indicated better growth, and the plants shown in the pictures were 14 days post-germination. Error bars represent the standard deviation of the means (*n* = 20). (**d**) All three *BocMBF1c*-OE lines showed a higher survival ratio after heat stress than WT (*n* =100). Error bars are the mean ± SD of three independent experiments. Photos were taken 2 days after heat stress. Asterisk indicates statistical significance by the Student’s test (*p* < 0.05).

We selected three T_4_ homozygous transgenic lines (Figure S3) overexpressed with the *BocMBF1c* and β-glucuronidase (*GUS*) fusion gene driven by the 35S *cauliflower mosaic virus* (*CaMV*) promoter (Figure 7a) for further study. We named them *BocMBF1c*–OE1, *BocMBF1c*–OE2, and *BocMBF1c*–OE3. β-glucuronidase activity analyses were performed with 5 day old seedlings to validate the expression of *BocMBF1c* (Figure 7b). We also examined the growth of 2 week old *BocMBF1c-*OE plants. The *BocMBF1c-*OE plants show larger sizes compared to wild type (WT) under normal growth conditions (Figure 7c) which is consistent with a previous report [14]. The five-day-old seedlings grown on MS plates were used for thermal tolerance analysis. After treatment at 46 °C for 2 h, the transgenic lines showed a survival ratio of 21.7% to 30.0%, while only 10.7% of the WT plants survived after heat stress (Figure 7d).

## 3. Discussion

### 3.1. BocMBF1c Can Respond to Multiple Stresses and Hormone Treatment in Addition to Heat Stress in Chinese Kale

We previously measured the effects of heat stress among 10 different Chinese kale varieties with different growth parameters, and the variety “Ai-jiao-xiang-gu” was found to be the most tolerable to heat stress [15]. We further measured the expression level of *BocMBF1c*; “Ai-jiao-xiang-gu” had the highest increase in expression level [15], indicating its important role in heat stress tolerance.

The promoter of *BocMBF1c* contained several HSEs; moreover, in *N. benthamiana* cells, the *BocMBF1c* promoter activity remained steady under ambient temperatures but was activated rapidly by high temperature stress. This is supported by semi-quantitative and qPCR evidence that the transcript abundance of *BocMBF1c* in planta is strongly and rapidly increased in response to heat stress treatment. Together, these data indicate that the expression of the *BocMBF1c* gene responds to heat stress via promoter activity. Our results showed that *BocMBF1c* can respond to cold, salinity, and several hormone treatments thus potentially possessing other functions besides thermal tolerance.

Bioinformatic analysis predicted that the *BocMBF1c* promoter contained ERE- and ABRE-responding cis-elements, implying ERFs and ABFs (ABRE binding factors)/ABEB could regulate the expression of *BocMBF1c*. Accumulation of ABA can initiate the expression of stress-responsive genes and participate in the regulation of the network through ABFs/AREB [16]. Ethylene and JA can stimulate the expression of downstream stress-related genes by activating ERF1 [17]. Previous reports showed that AtMBF1c functions upstream of SA and ET during thermotolerance [10]. From our results, *BocMBF1c* reacted distinctly under different hormone treatments. The expression levels of *BocMBF1c* both increased after exogenous application of MeJA and ABA although with different temporal response feature. For MeJA and ABA treatment, *BocMBF1c* expression peaked after 2 h and 4 h, respectively, which is possibly attributed to the difference in activation times for ERF1 and ABFs/AREB. After SA and ET application, the expression level of *BocMBF1c* both increased after a certain reduction period at the beginning. This indicates that *BocMBF1c* could possibly be negatively regulated by a high concentration of ET and SA, since the applied hormones would slowly degrade after application. With lower concentrations, ET can activate ERF1 thus stimulating the expression of *BocMBF1c*.

**Figure 8:**
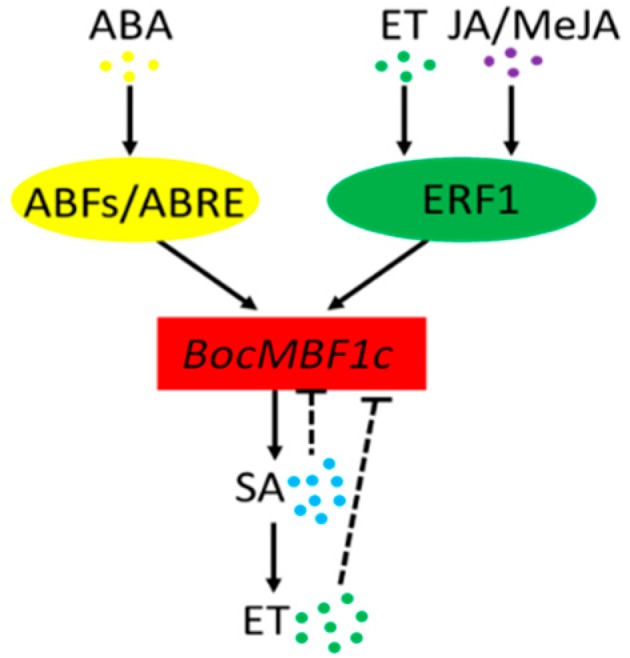
A model for the relation of *BocMBF1c* and other hormones. abscisic acid (ABA) and ethylene (ET) and jasmonic acid (JA)/MeJA activate ABRE binding factors (ABFs)/ABRE and Ethylene response factor1 (ERF1), respectively. ABFs/ABRE and ERF1 then upregulate the *BocMBF1c* gene by interacting with cis-elements in the promoter region. The BocMBF1c protein can regulate the salicylic acid (SA) and ethylene (ET) heat stress responsive pathway. High concentrations of SA and ET will inhibit the expression of *BocMBF1c* through negative feedback.

Overall, the accumulation of ABA, ET, and JA can activate corresponding transcription factors, which will then interact with the cis-elements located at the *BocMBF1c* promoter. The expressed BocMBF1c protein would then participate in the heat stress regulation network by regulating the downstream SA and ET signaling pathway (Figure 8).

Through analysis of the *Arabidopsis* public database (http://bar.utoronto.ca/efp_arabidopsis/cgi-bin/efpweb.cgi), we found *AtMBF1c* could be upregulated by heat, salt, and ABA treatment, and that it does not respond to cold and MeJA treatment (Figure S4) (http://bar.utoronto.ca/efp_arabidopsis/cgi-bin/efpWeb.cgi) [18]. The different responsive spectrums between *BocMBF1c* and *AtMBF1c* implies the *BocMBF1c* gene could participate in stress-related tolerance for the host plant.

The BocMBF1c protein quickly relocated to the nucleus when the cells were exposed to high temperature, further suggesting that this is a regulator for the expression of many heat stress-responsive genes. However, the mechanism of this nuclear translocation in a high-temperature environment remains unknown and should be the focus of future study. Likewise, downstream genes activated by this transcription factor remain unknown in Chinese kale, and transcriptome analysis by next-generation sequencing would be useful in the identification of candidate downstream genetic elements. Expression of these elements as well as known targets from other species can be further confirmed by qPCR. Earlier studies found the “CTAGA” element as a potential binding sequence for downstream genes; thus, it may be interesting to analyze the orthologs of the downstream genes in Chinese kale. This may reveal the reason for the relative temperature sensitivity of Chinese kale. Thus, it is highly likely that the *BocMBF1c* gene is necessary for multiple biotic/abiotic stresses in Chinese kale.

### 3.2. BocMBF1c Can be Applied to Improve the Productivity of Chinese Kale

The high-level conservation of *MBF1c* sequences (Table S1 and Figure S2) implies that this gene is critical for thermal tolerance and climate adaptation. For Chinese kale, bolting stems are the main edible tissue. The genetic mechanism underlying declined yield and quality of bolting stems under heat stress are still elusive in Chinese kale; cloning of *BocMBF1c* genes as a key heat tolerance factor would shed light on improving the heat tolerance of Chinese kale.

Transformation of Chinese kale is readily feasible and has been used for improving drought stress tolerance [19]. Our results indicate that the *BocMBF1c* gene can respond to heat treatment from both transcription and protein localization level. Overexpression *BocMBF1c* in *Arabidopsis* result in larger-sized plants with enhanced heat tolerance, while knockout of the *BocMBF1c* gene will be needed to fully validate its role in the thermal tolerance of Chinese kale, for example, by the CRISPR/Cas9-based gene editing technique [20]. Ectopic expression of *AtMBF1c* can improve tolerance to bacterial infection, heat, and osmotic stress in *Arabidopsis* [14]. Further study showed that overexpression of *AtMBF1c* could improve thermal tolerance without impairing the yield in *Arabidopsis* [10], soybean [11], and rice [21] under controlled conditions. A recent report showed that overexpression of *MBF1c* in Antarctic moss could make the plant bigger and enhance tolerance to salinity stress in *Arabidopsis* [12]. Thus, it will be intriguing to overexpress the *BocMBF1c* gene as a potential solution to improving the biotic/abiotic stress resistance and productivity of Chinese kale.

## 4. Materials and Methods

### 4.1. Plant Growth and Abiotic Treatment Conditions

We collected Chinese kale samples for tissue-specific expression analysis from the greenhouse (22–25 °C) at the South China Agricultural University (Guangzhou, China) [2]. For abiotic treatment, Chinese kale (*B*. *oleracea* var. *chinensis* Lei) variety “Ai-jiao-xiang-gu” were grown in a culture room under controlled conditions at 25 °C, with a 16 h light/8 h dark cycle. Chinese kale seedlings of 4~6 true leaves were transferred to the growth chamber for 24 h with the same conditions, and then the temperature was switched to heat (37 °C) and cold (4 °C) treatments. Leaf tissues were harvested for RNA extraction at 0 h, 0.5 h, 1 h, 2 h, 4 h, and 8 h.

For salinity treatment, each Chinese kale seedling of 4~6 true leaves were sprayed and watered by 100 mL of 100 mM NaCl, and leaf tissues were collected with the timeline of 0 h, 1 h, 2 h, 4 h, 8 h, and 16 h. Similarly, hormone treatments were carried out by applying 50 μM of MeJA solution, 100 μM ABA, 100 μM SA, and 100 ppm CEPA, respectively, and leaf tissues were collected along the same timeline. All samples used in this study were collected from three separated Chinese kale.

*Arabidopsis thaliana* (ecotype Columbia) were grown in chamber under controlled condition at 22 °C day/18 °C night, 70% relative humidity, and 100 μmol m^−2^ s^−1^ with a 16 h light/8 h dark light cycle. *Arabidopsis* seeds were germinated on MS plates or soil (Sunshine^®^ Mix #8, Sun Gro Horticulture) after being sterilized with 10% bleach for 10 min. All seeds were kept at 4 °C for at least 2 days before transfer to the growing chamber.

### 4.2. Cloning of the BocMBF1c CDS and Promoter

Leaves of Chinese kale were harvested for genomic DNA extraction [22]. We used the *MBF1c* gene from *Brassica rapa* (LOC103828628) as a reference sequence (Supplementary B1) to design primers for the CDS of *BocMBF1c*. The gDNA fragment containing the *BocMBF1c* CDS was amplified with primer MBF1c-up (5’-TCGAACTCTCCAGAAACTCGT-3’) and MBF1c-dw (5’-CGTTTGCCAGATACGATGT-3’). Next, *BocMBF1c* promoter was amplified with primer MBF1cP-F (5’-AGAATCCCCATTGACAGCCT-3’) and MBF1cP-R (5’-GGCATCGTCGCTGTTGTTTA-3’), designed from the gene sequence of *Brassica oleracea* (Supplementary B2). All PCRs were carried out with HiFiTaq PCR StarMix (GeneStar) according to the manufacturer’s protocol.

### 4.3. Sequence Analysis of the BocMBF1c Gene

The open reading frame (ORF) of *BocMBF1c* was predicted by the ORF finder (http://www.bioinformatics.org/sms2/orf_find.html), and the amino acid sequence was generated by the Expasy-Translate tool (https://web.expasy.org/translate/). Sequence query and alignment were carried out with BLAST (https://blast.ncbi.nlm.nih.gov/Blast.cgi). A phylogenetic tree was constructed with the software Mega 6.05, and the conserved sites were identified by DNAMEN.

The cis-elements of the *BocMBF1c* promoter were predicted by PlantCARE Database (http://bioinformatics.psb.ugent.be/webtools/plantcare/html/).

### 4.4. Promoter Activity and Subcellular Localization

The putative *BocMBF1c* promoter of the 1409 bp upstream sequence from the initiation codon was cloned into the binary vector pBI101-GFP with In-Fusion cloning, using primers designed following the manufacturer’s instructions (http://bioinfo.clontech.com/infusion/convertPcrPrimersInit.do), including pBI101 infusion M-P-F (5’-GCAGGTCGACTCTAGAGGTACCGAATAGCTCTCCCC-3’) and pBI101 infusion M-P-R (5’- CTTTACCCATTCTAGAGGCATCGTCGCTGTTGTTTA-3’). Similarly, CDS of the *BocMBF1c* was inserted into the pBE-eGFP construct by the In-Fusion technique, with primers MBF1-yxb–F (5’-CGCGGGCCCGGGATCCATGCCGAGCAGAT-3’) and MBF1-yxb-R (5’-GGCGACCGGTGGATCCTTGACATGTTT-3’). *Xba* I was used to digest the pBI101-GFP vector, and pBE-eGFP was digested by *Bam*H I, resulting in pBI101-*pBocMBF1c*-*GFP* and pBE-*BocMBF1c*-*eGFP* constructions.

Transient expression was carried out in the leaf tissue of *Nicotiana benthamiana* seedlings with 4 to 6 true leaves, and with *Agrobacterium* strain GV3101 harboring the pBI101-*pBocMBF1c*-*GFP* and pBE-*BocMBF1c*-*eGFP* constructs. The GFP signal was observed two days after agro-infiltration under fluorescent microscopy [23].

### 4.5. Gene Expression Patterns of BocMBF1c by qPCR and Semi-Quantitative RT-PCR

The RNA was extracted using HiPure Plant RNA Kits (Magen), and HiScript^®^ II Q Select RT SuperMix (Vazyme) was used for cDNA synthase. Gene expression was assessed by real-time PCR using AceQ^®^ qPCR SYBR^®^ Green Master Mix (Vazyme) and Bio-Rad iQ5 Multicolor Real-Time PCR Detection System. Primers MBF1c-YG1-UP (5’-GTTAACGCGGCTCTCAGAAG-3’) and MBF1c YG1-DW (5’-TCCTCCAGCTTCTTCGTGTT-3’) were used for *BocMBF1c*; *Tubulin8* was used as an internal control with primers Tubulin-F (5’-CTTCTTTCGTGCTCATTTTGCC-3’) and Tubulin-R (5’-CCATTCCCTCGTCTCCACTTCT-3’) [19]. The qPCR conditions: initial denaturation of 95 °C for 5 min, then 40 cycles of 95 °C for 10 s followed by 60 °C for 30 s; melting curves were obtained by gradually increasing the temperature from 60 °C to 95 °C for 15 s at a rate of 0.5 °C /s. Relative expression was calculated by the ∆∆Ct threshold method [24]. For heat and cold treatment, semi-quantitative RT-PCR was used to detect the expression pattern of *BocMBF1c* with Recombinant Taq DNA Polymerase TaKaRa Taq™ (Takara) and the same primers of qPCR. Semi-quantitative RT-PCR conditions: initial denaturation of 94 °C for 2 min, then 26 cycles for *Tubulin8* and 30 cycles for *BocMBF1c* of 95 °C for 30 s, 56 °C for 15 s, 72 °C for 10 s. Three biological replicates with triple technical replicates were used for all samples.

### 4.6. Arabidopsis Transformation and Phenotypic Analysis of Transgenic Plants

The *BocMBF1c* coding sequence was fused with *GUS* and then expressed in *Arabidopsis thaliana* driven by the *35S CaMV* promoter and then integrated into the *pBI121* binary vector. The floral dipping method [25] was used for *Arabidopsis* transformation. Transgenic seeds were selected on a 0.5 MS agar plate with 50 mg/L kanamycin and 300 mg/L timentin and then transplanted to soil for setting the seeds under standard growth conditions. Three homozygous transgenic lines from independent transformation events were used for further study (Figure S3).

Five-day-old *BocMBF1c*-OE and Col-0 *Arabidopsis* seedlings were collected for GUS staining analysis after germination on MS plates [13]. Two-week-old seedlings grown on soil were harvested for measuring fresh weight. For thermal tolerance analysis, five-day-old seedlings grown on MS plates were treated at 46 °C for 2 h, and then these plates were put back into growth chambers under normal condition for another 2 days.

## Supplementary Materials

Supplementary materials can be found at https://www.mdpi.com/1422-0067/20/22/5637/s1.

## Abbreviations

MBF	Multi-protein bridging factor
HTH	Helix-turn-helix
HSEs	Heat shock elements
GFP	Green fluorescent protein
eGFP	Enhanced green fluorescent protein
ABA	Abscisic acid
ABRFs	ABA responsive transcription factors
HSF	Heat stress transcription factor
HSPs	Heat shock proteins
MeJA	Methyl jasmonate
SA	Salicylic acid
ET	Ethylene
JA	Jasmonic acid
ERF	Ethylene response factor
DRE	Dehydration-responsive element
ORF	Open reading frame
CDS	Coding sequence
ERE	Ethylene-responsive elements
ABRE	Abscisic acid-responsive element
MBS	MYB binding site
N.	Nicotiana
qPCR	Quantitative PCR
CEPA	Ethephon
RT-PCR	Reverse transcription PCR
GUS	β-Glucuronidase
CaMV	Cauliflower mosaic virus
WT	Wild type
ABFs	ABRE binding factors
