# Supplementary material for: Cloning and Expression Analysis of the BocMBF1c Gene Involved in Heat Tolerance in Chinese Kale
Source: Int J Mol Sci. 2019 Nov 11;20(22):5637. doi: 10.3390/ijms20225637 (PMC6888671; doi:10.3390/ijms20225637)
Supplement: Supplementary file 1 [file ijms-20-05637-s001.pdf]

Supplementary A. Tables and Figures

Table S1. Homology analysis of the deduced amino acid sequence of *BocMBF1c* with other MBF1 proteins.

No.	Protein Name on NCBI	Origin	Coverage (%)	Identity (%)	Accession No.	Amino acids
1	BnaC07g07150D	*Brassica napus*	97	99	CDY39547.1	215
2	multiprotein bridging factor 1c	*Arabidopsis thaliana*	97	94	NP_189093.1	148
3	multiprotein-bridging factor 1c	*Arabidopsis lyrata subsp. lyrata*	97	94	XP_002883523.1	148
4	Multiprotein-bridging factor 1c	*Morus notabilis*	97	81	XP_010088841.1	147
5	multiprotein-bridging factor 1c	*Cucurbita maxima*	100	81	XP_022965996.1	145
6	Multiprotein-bridging factor 1c	*Morus notabilis*	97	81	XP_010088839.1	147
7	multiprotein-bridging factor 1c	*Cucurbita moschata*	100	80	XP_022937454.1	145
8	multiprotein-bridging factor 1c	*Vigna radiata var. radiata*	97	77	XP_014509028.1	145
9	Cro/C1-type helix-turn-helix domain	*Macleaya cordata*	97	76	OUZ99914.1	145
10	Helix-turn-helix type 3	*Corchorus capsularis*	97	76	OMO86847.1	143
11	multiprotein-bridging factor 1c	*Olea europaea var. sylvestris*	99	76	XP_022854816.1	146
12	multiprotein-bridging factor 1c	*Carica papaya*	97	75	XP_021903179.1	145
13	multiprotein-bridging factor 1c	*Prunus avium*	97	75	XP_021827691.1	144
14	Helix-turn-helix type 3	*Corchorus olitorius*	97	75	OMO58072.1	143
15	multiprotein-bridging factor 1c	*Arachis ipaensis*	97	75	XP_016200918.2	144
16	multiprotein-bridging factor 1c	*Arachis duranensis*	97	75	XP_015966499.1	144
17	multiprotein bridging factor 1	*Gossypium hirsutum*	100	75	AFN70435.1	145
18	HTH_3 domain-containing protein	*Cephalotus follicularis*	97	74	GAV79506.1	144
19	multiprotein-bridging factor 1c	*Prunus persica*	97	74	XP_007202731.1	144
20	ERTC family protein	*Populus trichocarpa*	97	74	XP_002324409.1	145
21	multiprotein-bridging factor 1c	*Manihot esculenta*	97	74	XP_021606733.1	144
22	multiprotein-bridging factor 1c	*Phalaenopsis equestris*	97	74	XP_020582421.1	148
23	multiprotein-bridging factor 1c	*Cajanus cajan*	97	74	XP_020233248.1	146
24	multiprotein-bridging factor 1c	*Jatropha curcas*	97	73	XP_012076664.1	144
25	Helix-turn-helix type 3	*Cynara cardunculus var. scolymus*	89	73	KVI01255.1	129
26	multiprotein-bridging factor 1c	*Spinacia oleracea*	97	72	XP_021838271.1	145
27	multiprotein-bridging factor 1c	*Hevea brasiliensis*	97	70	XP_021665665.1	144
28	multiprotein-bridging factor 1c	*Dendrobium catenatum*	98	70	XP_020699739.1	148
29	ethylene-responsive transcriptional coactivator	*Hevea brasiliensis*	97	70	AGQ57014.1	144
30	multiprotein-bridging factor 1c	*Helianthus annuus*	97	68	XP_022027306.1	145
31	Multiprotein-bridging factor 1c	*Capsicum chinense*	100	68	PHU17242.1	143
32	ethylene-responsive transcriptional coactivator	*Solanum lycopersicum*	97	66	AAD46402.1	146

No.	Protein Name on NCBI	Origin	Coverage (%)	Identity (%)	Accession No.	Amino acids
33	ethylene-responsive transcriptional coactivator	*Solanum lycopersicum*	97	66	NP_001234468.2	146
34	ERTC	*Populus tomentosa*	97	66	AGM20673.1	218
35	multiprotein-bridging factor 1c	*Sorghum bicolor*	99	65	XP_002438624.1	155
36	multiprotein-bridging factor 1c	*Ananas comosus*	98	64	XP_020088046.1	144
37	multiprotein-bridging factor 1a	*Arabidopsis thaliana*	95	48	AEC10155.1	142
38	multiprotein-bridging factor 1b	*Arabidopsis thaliana*	95	51	CP002686.1	142

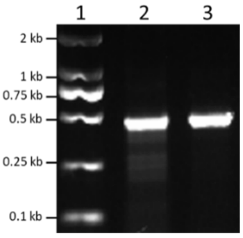

Figure S1. The results of the coding sequence (CDS) amplification of *BocMBF1c*. **1**. The channel of Maker, M2000, was used as a DNA ladder, yielded bands of 2 kb, 1 kb, 0.75 kb, 0.5 kb, 0.25 kb, and 0.1 kb from up to the bottom in the lane; **2**. and **3**. amplicons of *BocMBF1c* CDS from gDNA and cDNA, respectively.

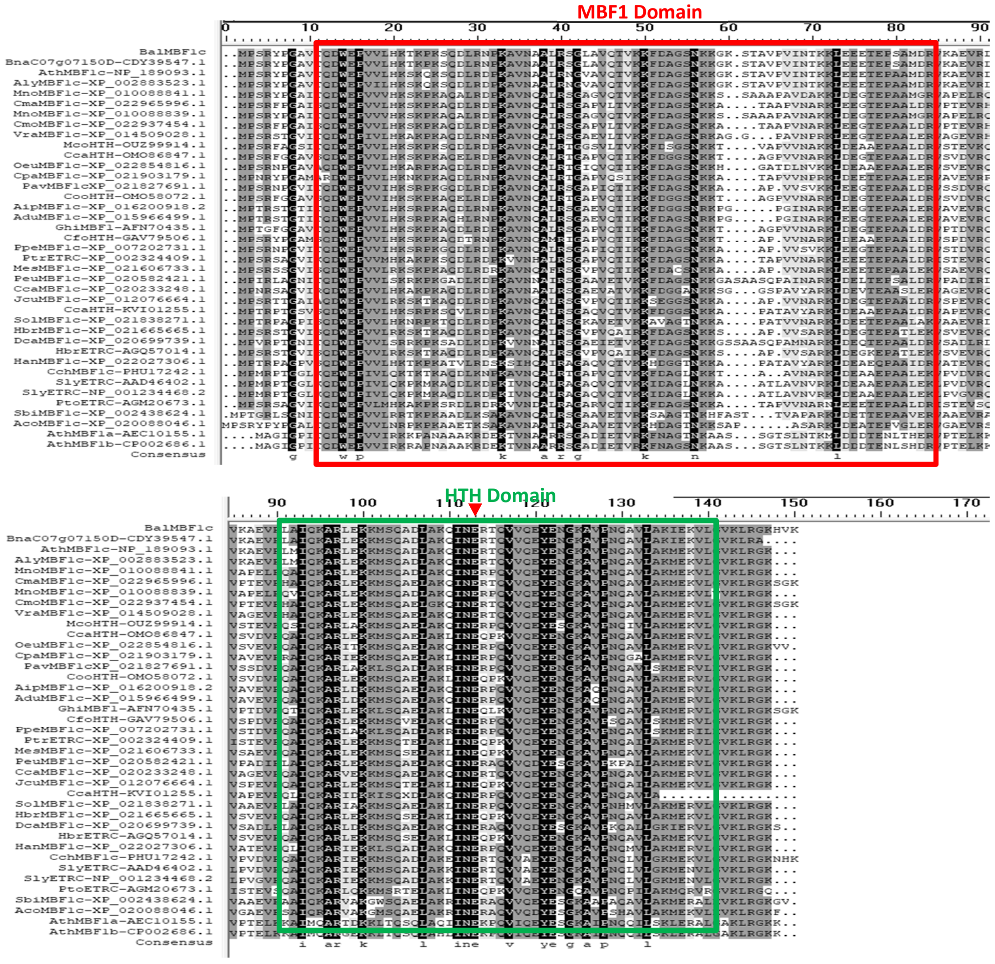

Figure S2. Multiple alignments of the predicted amino acid sequence around the conserved domain of MBF1 and HTH from *BocMBF1c* with various other similar proteins. The 38 proteins used in this

program are listed in Table S1. Gaps are indicated by a solid circle; conserved amino acids are shaded black and similar amino acids are shaded gray. Red underlines indicate the *MBF1* gene family domain; green underlines indicate the HTH domain.

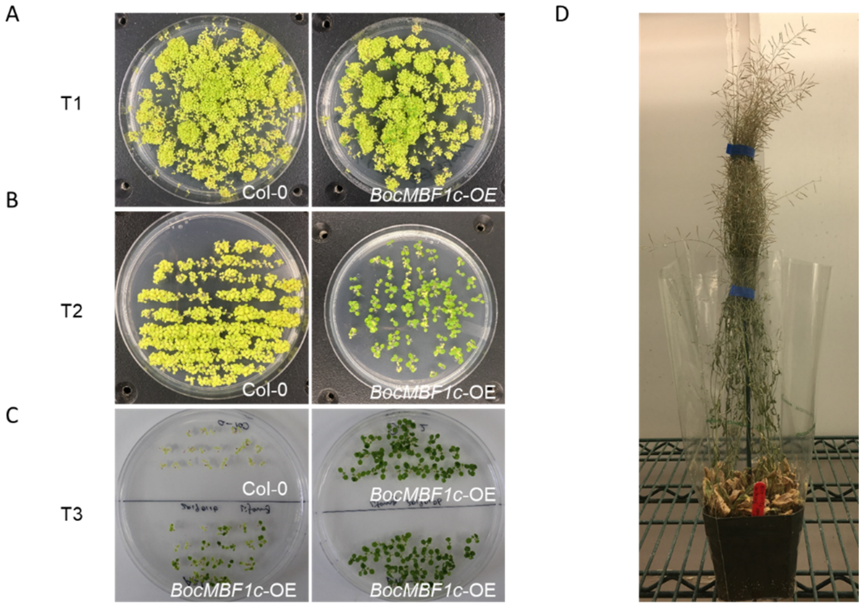

Figure S3. The selection of homozygous transgenic lines of *BocMBF1c*. (**A**) The T_1 seeds that survived the Kanamycin selection were collected as positive transgenic lines; (**B**) the T_2 generation showing segregation of resistant-to-sensitive under Kanamycin selection; (**C**) the T_3 generation showing 100% resistance to Kanamycin were chosen as homozygous transgenic lines for further study; (**D**) the T_4 seeds that will be mix harvested from T_3 generation.

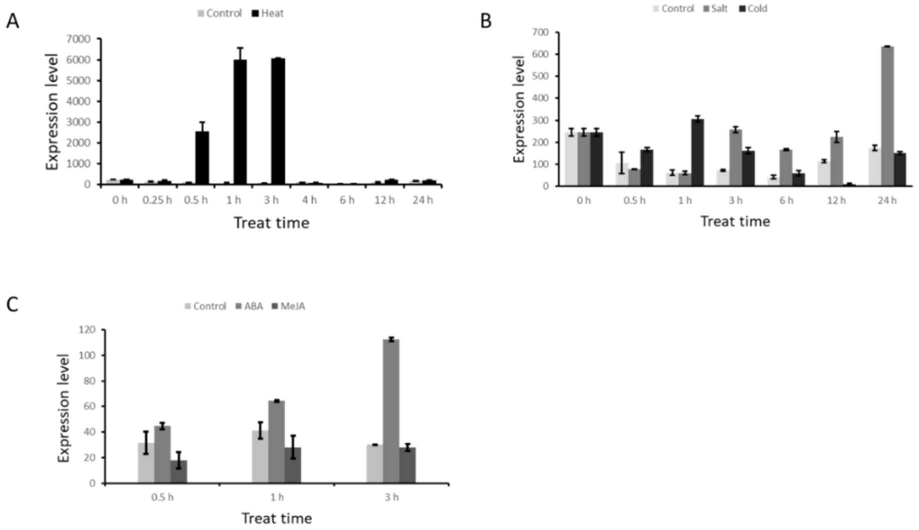

Figure S4. Response of *AtMBF1c* to various abiotic treatments. (**A**) Temporal expression of *AtMBF1c* under heat treatment (30 °C). (**B**) Temporal expression of *AtMBF1c* under cold condition (4 °C) and salinity environment (200 mM NaCl). (**C**) Temporal expression of *AtMBF1c* after applied ABA (10 μM), MeJA (10 μM), respectively. All data were obtained through an online database (http://bar.utoronto.ca/efp_arabidopsis/cgi-bin/efpWeb.cgi) [18].

Supplementary B. Supplementary Sequences

B1. Sequence of the *BrMBF1c* Gene

AAAAAGTATAAATGAATAAAAATAATAGTAGCTGCAAAAAAAAGTTTTCAAAAAAAAAA
TTAATACCATAAACAAAACACTAAACTCTAAATCTAAACCCTAAACTCTTTGGTAAACCCT
AAACCCTTTGATAAATTTTAAATTCTTGAATTTAAAAAAAAATATTTTTAATACAATCAACA
AAACACTAAATCCTAAAACCTAATCCTAAACCCTAAACCCTTGGGTAAATCCTAAAAAAA
ATTCATTTTTTTGCGATTAAAAAATCTTCTTTTTGTGATTAATACAATTTTTATTTTAAAAATA
TAATATAGTTCGATAATATTTTGTTTCCTTTTTTAAAAGATATCAGATTTAAAATAACTAAAT
TTTTCTTATTGGTTGGTGAACCTACAGGTTCCCAAGAATAAGTCTTTCTATTACTGTCTACAG
TTGGATACAAGACATAAATTTATAGTAACGCCACGTCACCTACAATAACACCCAATAAAAC
AAGAACACGTCACCAGCCTAAGAACTGTCCAGAAACGAAAATGGAGTGATCGAACTCTCC
AGAAACTCGTCTCCTTATATAAAGACCTCTCCTCCCAGCGACGCCTTCATCGTTCTCAATTT
CAGAAACTCATCATTATCATCATCTCTCTGAGATATCAAGTTTTAAACAACAGCGACG**ATG**
CCGAGCAGATACCCAGGAGCCGTGACGCAAGACTGGGAGCCAGTGGTGCTCCACAAAACC
AAGCCAAAGAGCCAAGACCTCCGTGATCCCAAGGCGGTCAACGCAGCTCTCAGAAGCGGC
TTAGCGGTTCAGACTGTGAAGAAATTCGACGCAGGTTCGAACAAGAAGGGCAAATCGATG
GCGGTGCCGGTGATCAACACGAAGAAGCTGGAGGAAGAGACGGAGCCGTCGGCGATGGA
TAGGGTGAAAGCGGAGGTGAGGCTAGCGATACAGAAAGCTCGGTTGGAGAAGAAGATGTC
ACAAGCGGATCTAGCGAAACAGATCAACGAGCGGACACAGGTGGTTCAAGAATACGAAA
ACGGGAAAGCTGTTCCTAACCAGGCCGTGCTTGCCAAGATGGAGAAGGTTCTTGGTGTTAA
ACTCAGGGGTAAACATGTCAAA**TAA**TTCAAAACGATGCCGTCTCTGGTTTCGTTCTTCCTTC
TTTTGGGTGCACGTCTAATTTCGTATTCTCAATCTTATGAATATAAATAATAACGCTTTTGGC
TTCGTATTCAAACGACATCGTATCTGGCAAACGATTGTCAAATTTGAAAAGGAAGTGGGTT
TGTTCTGTTTGATTTAACTTGAATTTCTTCATACATGAGTCATGACTGGTCTAAATATTTCTTA
TTTGCATGTATAATTGTATTCCTGTAATCAACAAGATTAATAATCCTTTTTTAAAACAAAGTC
GGTGTTTTATGGACATCTCTGGACTTGGTCTTATAGGTCTAATGGGTTTGTGACTCAGAACTT
CTTGGTTATCATATAAATTTAGAAATGAATCAAGAAATGTGTAATGGTAGCGTGACATCTG
CATCTGTGAGTAAGTTACTCGGTTCGATGAAGCATCCTTTGTTTCTAAGCGGGAGTCCAAAC
TAGACAGAATCAGTATTTGTAACATGTTTAGATTTTTTAAGTACATTAAATACAACGAAAAG
TAAGCAGAAAGAATGCTCAAACAATTTGATAGGTAAAAGTTTAAAACTTTATTTCAAATTT
GCTAACGTTTAAGAGACGAAACCATTTAGCAAACTGAAAAGATTGTAACTTGGTACTCCCT
TACCTACACGTCACGATCCCTCTTTT

Start code and stop code were in bold. Primers designed from BrMBF1c gene, including MBF1c-up and MBF1c-dw were marked in red.

B2. Promoter Sequence of the *BoMBF1c* Gene

TTGCAAAGGCAGCACCAAAACTCTTCAGTTCTGTTGCCATACGGGAAACTTCTGAGGAAAC
ACGGTTCAGCTCTTCTGATACACGACTCAGATCTCCTGAAAGGCGACCTTTTACACAACTAG
CTATGGAATCTGCATCCACAACATCACCACTACTCTGGTTTGATTTCTGCTTTGTTGTTTTCCT
GATCTCTGCTTCACTTCTTGTTTCTTCCCGCATTCTTACAACATCTGATTTTGAAACACCTCCC
GTAAAAAAAAGAATGCTTGAATGGAAATCCATCGTCGATTAACTTTAGCAGATTTTGTACA
CCGACATCTTCTTCATCATCAGACCACCCAAACTGAGAATCCCCATTGACAGCCTCGTTCCC
AACCGAAATAATGGAAACCACCCCGGCCTGCCGTTGAAATTGATGTCAGTCAAGATTACA
ATAACATATTATAGCAGATGTTATTTCCTACTATATTACCTTTCCAGAACAGTCAGTATCA
CGGGCATGCCCTGGACTTATACTTCGCTTCCCTTTTCTTTCTTCTTCTGAAAGTTCGTCGTCGT
CCGCAAAATCGCCTTCCGAACCAGACGAGCCGGCTTCATGAACGGCTTCGGTAAGAGCAG
GTACAGCTTCAATCATTACAAGCTGTAAAGCTAGCACAAATCCCTTTAGAGCTATTGTGTTT
TGGGATAGAGAAAGTTTGTTCCTCTCTTTGATACTGCTTATAAGCAACGCGACCCCAAGGAT
ATGCAAGAAACGCATCCAAATCTTTAATCTTCTCCGCATGTTCGTGTGAAATCCGAGGTGTA
TGAGTTGTTGGGAGAACAACAGATGCAAGGAGTGCAAGGAACGCATACTTAATCCTTGTTT
CTTTTGCAGTTACACTTCTCTTTTTCAACATCCTTACAACACAGTTGGCAGATACTTCTTTCA
TGGTACTGAATATGGCAAATTCCCTCAGAGAAAATCGGATTGGTTTGCCTGCAAACAAGAA
CCATGCTTCATGTTTCTTCTTCACCTTCAATTGCCTCGATATAATATATCTCCCAAAACGTCC

TGAGAATGTTGGTTTCTCCGCGATTTCTACAAGCTTACCGAACGGTGATCGGCGCAGAGCAT CGACTTCATCTTCTTCCAGTGATCGGCGCAGAGCATCGACTTCATCTTCTTCCAGTGACCGG AGTATGTTACTTATTGCGAAGGGTTTGTGGTAAGGGGTAACTCTCACCTCCACGGGTTCCTC GCCCAAAGCAAACATCCTCTCCGGTAATGTTCGCGAAGACTCCATATCTGCTCGATTTTCCA TCTCCATCGTTGGTACCGATGTCATCAATCAAAGAAGATGAGCAAAAAGCTTAGGGAGAG AGTTTCCAGATTTGAGACAAGTTCTACAGAGAAGGTCCATTTTAGAGATGATTTCAAATTTC GAAATACAAAATTGAAATTACAAAGACATATAATCTTTCAAGATTTGGGTTTTCTGACACA AAATACGAAGATACGAAAATATTTCAAATATCATGGATACGCAAATCTTTGTAAATCTTAA CCAATAGATTACATAATCTTTTAGGACGCAGACGCATGGGTGATATCGATAGGGGTAGCGC TGTCTTTACCATCTGATGAACAGTTGCAATTAGCTCGAATTGGTATATGCCTAAATACGCAA TATGTTGAGTGCAGGGAGTGCAATTTCACTAATATGTATTGCAGTATTTAGTTTGTTTGTTCT GGATTGCTCATTTACCATTCGAAGCCTAAAGCATCATTGGCTTTGCATTTTTCGACTCTTCAC GCTAGAGGTCCTAAGGGCATCATTAACCCAGGTCTCTTAGCCGGAGTTTTTAACTCATGATT TGATATTTTTTTGTTTTTTTTTTACATTTTTTTTACTAAAAAACATCTCTTATATCTCTTATTTA AGAGACGGTTCTTAGTCGAACCTAAGAATTTCAGTTAAGAGACTGGGGTTAGTGATCGTCG GACGAACGTGAGATACTTTTCTATTACTGTCTACCATTAGATAGAAGACGAGTTCATTTAGA TAAATTTATAGTGACGCCACGTCACCTGCAATAACACCCAATAAAACAAGAACACGTCAC CAGCCTAAGAACTGTCCAGAAACGAAAATGGAGTGATCGAACTCTCCAGAAACTCGTCTC CTTATATAAAGACCTCTCCTCCCAGCGACGCCTTCATCGTTCTCAATTTCAGAAACT. Primer MBF1cP-F is in red.
